# Epicardial Fat Thickness as a Marker of Coronary Artery Disease Severity and Ischemic Burden: A Prospective Echocardiographic Study

**DOI:** 10.3390/jcm15020657

**Published:** 2026-01-14

**Authors:** Dafni Charisopoulou, Sotiria Iliopoulou, George Koulaouzidis, Nikolaos Antoniou, Kyriakos Tsantekidis, Aggeliki D. Mavrogianni, Michael Y. Henein, John Zarifis

**Affiliations:** 1 Cardiology Department, General Hospital George Papanikolaou, 57010 Thessaloniki, Greece; sotiria.ili@gmail.com (S.I.); nikosn771@gmail.com (N.A.); kktsante1@gmail.com (K.T.); amavrogianni@yahoo.com (A.D.M.); zarifis.john@gmail.com (J.Z.); 2 Department of Biochemical Sciences, Pomeranian Medical University (PMU), 70-204 Szczecin, Poland; koulaou@yahoo.co.uk; 3 National Heart and Lung Institute, Imperial College, London SW3 6LY, UK; henein@gmail.com

**Keywords:** epicardial fat thickness, coronary artery disease, myocardial ischemia, transthoracic echocardiography, dobutamine stress echocardiography, cardiovascular risk stratification

## Abstract

**Background/Objectives**: Epicardial fat thickness (EFT) is an echocardiographic marker of epicardial adipose tissue that has been linked to coronary atherosclerosis, but its relationship with both coronary artery disease (CAD) severity and myocardial ischemia remains incompletely assessed. This study evaluated the association between EFT, angiographic CAD severity, and stress-induced myocardial ischemia. **Methods**: In a prospective study, 125 consecutive patients with suspected stable angina underwent transthoracic echocardiography with EFT measurement, dobutamine stress echocardiography, and coronary angiography. EFT was measured at end-systole in the parasternal long-axis view. Significant CAD was defined as ≥50% stenosis in at least one major epicardial coronary artery. Myocardial ischemia was assessed using peak-stress wall motion score index (WMSI). **Results**: Significant CAD was present in 56% of patients. Mean EFT was significantly higher in patients with significant CAD compared with those without (7.8 ± 2.0 mm vs. 5.5 ± 1.5 mm; *p* < 0.001). EFT increased progressively with angiographic CAD severity (non-significant CAD: 5.5 ± 1.5 mm; one-vessel disease: 6.5 ± 1.8 mm; two-vessel disease: 7.5 ± 2.0 mm; three-vessel disease: 8.5 ± 1.9 mm; *p* < 0.001). Patients with EFT > 5 mm had a significantly higher prevalence of significant CAD (68.8% vs. 33.3%; *p* < 0.001) and were older, with higher body mass index and a greater prevalence of hypertension and obesity. Additionally, peak-stress WMSI was significantly higher in patients with elevated EFT (1.08 ± 0.07 vs. 1.04 ± 0.05; *p* = 0.005), indicating a greater ischemic burden. **Conclusions**: EFT is associated with both the anatomical severity of CAD and the extent of stress-induced myocardial ischemia, supporting its potential role in non-invasive risk stratification of patients with suspected CAD.

## 1. Introduction

Epicardial adipose tissue (EAT) is a metabolically active visceral fat depot situated between the myocardium and the visceral layer of the pericardium [1,2,3,4,5]. It envelops most of the heart’s surface, particularly along the atrioventricular and interventricular grooves, the right ventricular free wall, and the coronary arteries [1,2,3,4]. Embryologically, EAT originates from the mesoderm, sharing its developmental pathway with intra-abdominal visceral fat, and is primarily derived from brown adipose tissue [5,6]. It constitutes approximately 20% of the total ventricular mass and covers up to 80% of the cardiac surface. The lack of a fascial barrier between the myocardium and epicardial fat allows a shared microcirculation through the coronary arteries, promoting continuous molecular and metabolic cross-talk between these two tissues [1,2,3,4].

Coronary artery disease (CAD) remains a leading cause of morbidity and mortality worldwide and represents the clinical manifestation of progressive coronary atherosclerosis. Its development is strongly influenced by established cardiovascular risk factors, including age, obesity, hypertension, diabetes mellitus, dyslipidemia, and smoking. These factors contribute to systemic inflammatory and metabolic disturbances that promote both vascular disease and adipose tissue dysfunction.

Uncertainties remain regarding the exact role of epicardial adipose tissue in CAD. While several studies have demonstrated an association between epicardial fat and CAD severity, a direct relationship has not been established. Epicardial fat accumulation and CAD may instead represent parallel manifestations of shared pathophysiological processes, including systemic atherosclerosis and common cardiovascular risk factors [7,8,9,10,11,12,13,14,15,16,17]. This study seeks to investigate the association between EFT, studied by echocardiography, and significant CAD, with severe stenosis defined as ≥50% stenosis on conventional angiography. The study also explores potential relationships between EFT and myocardial ischemia assessed by stress echocardiography and established cardiovascular risk factors.

**Figure 1:**
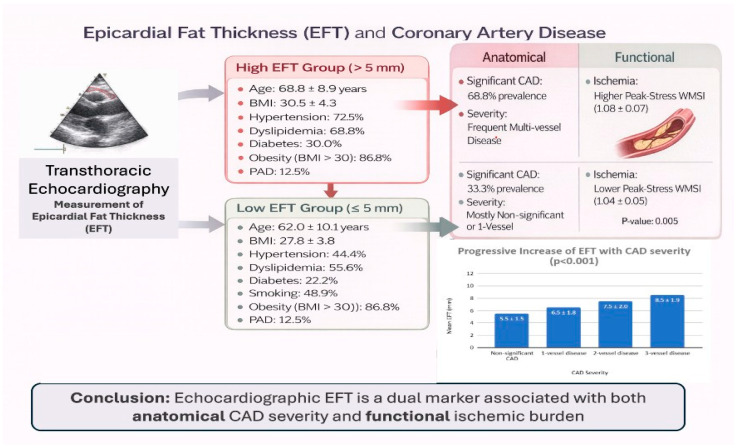
Schematic overview of the study design and main findings. The figure illustrates the study workflow, including patient selection, assessment of epicardial fat thickness by transthoracic echocardiography, evaluation of myocardial ischemia by dobutamine stress echocardiography, and anatomical assessment of coronary artery disease severity by coronary angiography. The main functional and anatomical associations observed in the study are summarized.

Figure 1 provides a schematic overview of the study design and summarizes the main anatomical and functional associations observed between epicardial fat thickness, coronary artery disease severity, and stress-induced myocardial ischemia.

## 2. Materials and Methods

This is a prospective observational study conducted at Thessaloniki Heart Center, a single tertiary center. Consecutive patients with angina pectoris who were referred for assessment of CAD were considered eligible for inclusion in the study. The study was approved by the institution ethics committee (reference number 665) and was conducted in accordance with the Declaration of Helsinki.

Patients were considered eligible for inclusion in the study based on predefined criteria, including age ≥ 18 years, regardless of sex, with a clinical presentation and history suggestive of stable angina (mainly chest pain, breathlessness or both). Patients were excluded from the study if they exhibited any of the following criteria: presented with acute coronary syndrome (ACS), known history of CAD, previous percutaneous coronary intervention (PCI), prior coronary artery bypass graft (CABG) surgery, more than mild valve disease and heart failure with reduced EF, or if they have poor echocardiographic images. Patient recruitment was based exclusively on clinical presentation and referral for investigation of suspected stable angina and was not influenced by body mass index or adiposity-related characteristics.

All patients underwent a thorough medical history review, capturing demographic details including age, sex, and body mass index (BMI). Key cardiovascular risk factors, such as smoking status, diabetes mellitus, hypertension, dyslipidemia, overweight/obesity and family history of premature CAD, were systematically evaluated.

The presence of risk factors was confirmed using referral letters and patient questionnaires. A family history was defined as CAD in at least one first-degree relative (men < 55 years and women < 65 years). Diabetes mellitus was defined as fasting plasma glucose level ≥ 126 mg/dL (7.0 mmol/L), current use of hypoglycemic medications, or a self-reported prior diagnosis of diabetes. Hypertension was defined as the use of antihypertensive medications or a known but untreated hypertension. Hypercholesterolemia was defined as the use of cholesterol-lowering medications or a known but untreated total serum cholesterol level > 240 mg/dL (6.2 mmol/L). Current smokers and individuals who had quit smoking within 30 days prior to the study were classified as smokers, while all others were considered nonsmokers. Obesity was defined as a body mass index (BMI) ≥ 30 kg/m^2^.

Any relevant comorbidities were also documented, e.g., peripheral arterial disease or stroke. Each patient received a comprehensive cardiac examination and a resting electrocardiogram (ECG). Data were recorded using a standardized, pre-designed form, with strict adherence to patient confidentiality, ensuring all information was used exclusively for research purposes.

### 2.1. Transthoracic Echocardiography

**Figure 2:**
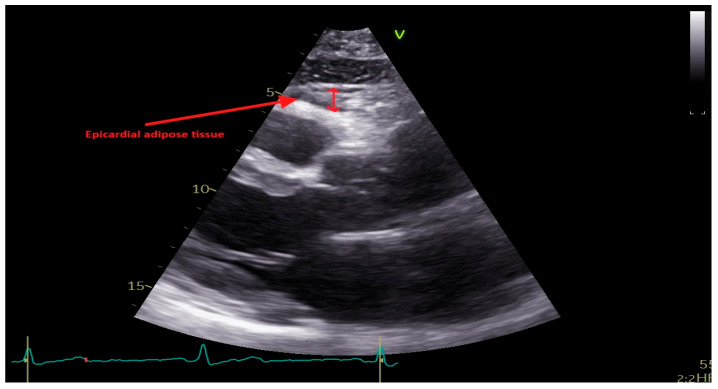
Measurement of epicardial fat thickness by transthoracic echocardiography.

Standard two-dimensional transthoracic echocardiograms (TTE) were performed with patients positioned in the left lateral decubitus position. Imaging was conducted using a commercially available ultrasound system (Vivid E95, General Electric Vingmed Ultrasound, Milwaukee, WI, USA) equipped with a 3.5 MHz or M5S transducer. Two experienced echocardiologists performed the examinations, adopting the chamber quantification recommendations of the American Society of Echocardiography and the European Association of Cardiovascular Imaging, as well as expert consensus guidelines from the latter [18,19]. All acquired echocardiographic images were digitally stored and analyzed offline using EchoPac version 12 (GE Healthcare, Chicago, IL, USA) by two investigators blinded to baseline clinical and angiographic data. Left ventricular end-diastolic and end-systolic volumes were calculated using Simpson’s biplane method from apical two-chamber and four-chamber views, and left ventricular ejection fraction (LVEF) was calculated. EFT was measured in the parasternal long-axis view at end-systole, along the right ventricular free wall, perpendicular to the aortic annulus, and recorded in millimeters, Figure 2.

Epicardial fat thickness (EFT) is measured at end-systole in the parasternal long-axis view as the echo-free space between the outer myocardial wall of the right ventricle and the visceral layer of the pericardium.

### 2.2. Dobutamine Stress Echocardiography (DSE) Assessment

Participants stopped β-blockers for ≥48 h before DSE. A cardiologist assisted by a nurse conducted the study; both were blinded to all patients’ clinical details. After obtaining resting images to rule out significant valvular disease, intravenous dobutamine infusion was started at 10 μg·kg^−1^·min^−1^ and increased to 20, 30, and up to 40 μg·kg^−1^·min^−1^ in 3 min stages. Patients not reaching 85% age-predicted heart rate were given atropine in 300-μg increments to a maximum of 1200 μg. Images were acquired from the apical 2-, 3-, and 4-chamber views, in the parasternal long-axis and short-axis view at baseline, low-dose stress, peak (high-dose) stress, and recovery. In all cases, echo contrast, SonoVue (Bracco Imaging SpA, Milan, Italy), a commercially available sulfur hexafluoride microbubble preparation was used. This was given as 0.3 mL intravenous boluses for each image acquisition.

Image interpretation was performed by two independent blinded observers. In cases of disagreement, a third observer was consulted. Regional wall motion and systolic wall thickening were scored using a standard 16-segment LV model. Each segment was scored using a 5-point scale as follows: 1 = normal, 2 = mild hypokinesis, 3 = severe hypokinesis, 4 = akinesis, and 5 = dyskinesis. Myocardial ischemia was defined as new or worsened wall motion abnormalities during stress, indicated by an increase of ≥1 point scale in ≥1 segment of the wall motion score [20,21,22]. A biphasic response in an akinetic or severely hypokinetic segment was considered as an ischemic response. An akinetic segment, at rest, that became dyskinetic during stress, was taken as a manifestation for ischemia [20,21,22]. The wall motion score index (WMSI) was calculated by dividing the sum of segment scores by 16. The WMSI was obtained at rest and at peak stress [20,21,22].

### 2.3. Coronary Angiogram

All patients with positive DSE examination received a coronary angiogram with revascularization, if needed and if feasible. Coronary angiograms were undertaken adopting the conventional Judkins technique. Angiographic images were obtained in multiple projections for each coronary artery. The extent of coronary artery stenosis was visually estimated by an experienced operator who was blinded to the echocardiographic findings. A luminal narrowing of ≥50% in any of the major coronary arteries, including the left anterior descending artery (LAD), left circumflex artery (LCX), and right coronary artery (RCA), was considered significant stenosis.

### 2.4. Statistical Analysis

Data were analyzed using Python 3.12 (NumPy, SciPy, Pandas, Matplotlib). Normality was assessed with the Shapiro–Wilk test; non-parametric tests were applied for skewed distributions (EFT, WMSI, BMI). Descriptive statistics included means ± standard deviations for continuous variables and frequencies for categorical variables. The primary objective of the data analysis was establishing an association between EFT and CAD severity, using Spearman correlation, Kruskal–Wallis’s test, and chi-square test for categorical EFT. Secondary objectives included identifying associations between the following:

- EFT and myocardial ischemia, using Mann–Whitney U test, Spearman correlation with post-stress WMSI, and chi-square test.
- EFT and EF, using Spearman correlation, Kruskal–Wallis’s test across EF groups (<40%, 40–50%, >50%), and linear regression adjusted for age and sex.
- EFT and risk factors, applying Mann–Whitney U tests, Spearman correlation with BMI, chi-square tests, and multiple regression adjusted for age, sex, and all risk factors.

A *p*-value < 0.05 was considered statistically significant.

## 3. Results

### 3.1. Study Population

The study population consisted of 125 consecutive individuals who fulfilled the inclusion and exclusion criteria, mean age was 66.2 ± 9.8 years, and the majority (90 subject; 72%) were males. Overall, 34 subjects (27.2%) were diabetics, 78 (62.4%) had hypertension, 45 (36%) were obese, and 60 (48%) smoked. A total of 80 subjects (64%) had dyslipidemia, and 15 (12%) had peripheral arterial disease. Echocardiographic mean LVEF was 55.0 ± 7.5%, and mean EFT was 6.9 ± 2.3 mm.

### 3.2. EFT Group Comparison (≤5 mm vs. >5 mm)

Of the 125 participants, 80 patients had EFT > 5 mm (high EFT) and 45 had EFT ≤ 5 mm (low EFT). Risk factors comparison of these two groups revealed significant differences, Table 1. Patients with EFT > 5 mm were older (68.8 ± 8.9 vs. 62.0 ± 10.1 years, *p* = 0.001) and had a higher body mass index (BMI) (30.5 ± 4.3 vs. 27.8 ± 3.8, *p* = 0.002). Hypertension was more prevalent in the EFT > 5 mm group (72.5 vs. 44.4%, *p* = 0.002), as was obesity (BMI > 30) (86.8 vs. 48.0%, *p* = 0.001). However, no significant differences were observed in the prevalence of dyslipidemia, diabetes, smoking, or peripheral artery disease between the two groups.

### 3.3. Significant CAD vs. No Significant CAD

Significant coronary artery stenosis (CAD), defined as >50% stenosis in one or more coronary arteries, was present in 70 subjects (56%). The remaining 55 subjects (44%) had ≤50% stenosis. CAD severity was distributed as follows: single-vessel disease was present in 30 subjects (24%), two-vessel disease in 29 (23.2%), and three-vessel disease in 11 (8.8%).

Patients with significant CAD were older (67.8 ± 9.1 vs. 64.0 ± 10.2 years, *p* = 0.024) and had a higher prevalence of hypertension (71.4 vs. 50.9%, *p* = 0.017) and dyslipidemia, though did not reach statistical significance (71.4 vs. 54.5%, *p* = 0.052) compared with those with non-significant CAD, Table 2. They also had lower LVEF (52.8 ± 7.1 vs. 55.5 ± 6.2%, *p* = 0.022). No significant differences regarding BMI, diabetes, smoking status, or PAD were observed between groups.

In this cohort of 125 patients, there was a significant association between EFT and CAD. Patients with significant CAD exhibited markedly higher mean EFT than those without CAD (7.8 ± 2.0 vs. 5.5 ± 1.5 mm; *p* < 0.001). Mean EFT increased progressively with increasing CAD severity (Table 3) from 5.5 ± 1.5 mm in patients with non-significant CAD to 6.5 ± 1.8 mm with one-vessel disease, 7.5 ± 2.0 mm with two-vessel disease, and 8.5 ± 1.9 mm with three-vessel disease (*p* < 0.001). Post hoc analyses confirmed significant differences between non-significant CAD and all vessel disease groups (*p* = 0.045 for non-significant CAD vs. 1-vessel; *p* < 0.001 for non-significant CAD vs. 2-vessel; *p* < 0.001 for non-significant CAD vs. 3-vessel), underscoring a clear trend of increasing EFT with CAD severity.

In individual patients with low-EFT, 15 (33.3%) had significant CAD, compared with 55 (68.8%) in the high-EFT group (*p* < 0.001). Also, the distribution of CAD severity differed significantly between the two groups (*p* < 0.001). In the low-EFT group, 30/45 (66.7%) had non-significant CAD, 10/45 (22.2%) had one-vessel disease, 4/45 (8.9%) had two-vessel disease, and 1/45 (2.2%) had three-vessel disease. Respective values in patients within the high-EFT group were 25/80 (31.3%) with no-significant CAD, 20/80 (25.0%) with one-vessel disease, 25/80 (31.3%) with two-vessel disease, and 10/80 (12.5%) with three-vessel disease.

### 3.4. EFT and Myocardial Ischemia

The relationship between EFT and DSE findings was also analyzed by comparing patients with low EFT (≤5 mm, n = 45) and high EFT (>5 mm, n = 80). A positive DSE, for ischemia, was found in 15 patients (33.3%) in the low-EFT group compared to 35 patients (43.8%) in the high-EFT group, though this difference was not statistically significant (*p* = 0.259). The mean WMSI at rest was slightly higher in the high-EFT group (1.05 ± 0.05) compared to the low-EFT group (1.04 ± 0.04), but this difference was also not statistically significant (*p* = 0.323). However, the peak-stress WMSI was significantly higher in the high-EFT group (1.08 ± 0.07) compared to the low-EFT group (1.04 ± 0.05, *p* = 0.005), indicating worse wall motion abnormalities in patients with greater EFT.

The mean number of myocardial segments with wall motion abnormalities at rest was slightly higher in the high-EFT group (1.1 ± 1.9) compared with the low-EFT group (0.8 ± 1.7); however, this difference was not statistically significant (*p* = 0.412). A similar numerical trend was observed at peak stress (*p* = 0.078), with a greater number of abnormal segments in the high-EFT group, although this did not reach statistical significance.

## 4. Discussion

In this prospective echocardiographic study, EFT was significantly associated with both the presence and severity of CAD, as well as with the extent of stress-induced myocardial ischemia. EFT increased progressively with the number of diseased coronary vessels and was associated with higher peak-stress wall motion score index, indicating a greater ischemic burden. These findings support the value of EFT as a readily obtainable imaging marker associated with both anatomical and functional manifestations of coronary artery disease.

EAT is a metabolically active visceral fat depot located in direct anatomical proximity to the coronary arteries [23]. Under physiological conditions, EAT may exert protective effects; however, in the presence of obesity and metabolic dysfunction, it may adopt a pro-inflammatory phenotype [24,25]. Through local paracrine and vasocrine signaling, EAT has been proposed to contribute to coronary atherosclerosis and myocardial dysfunction [26]. Nevertheless, epicardial fat accumulation and coronary artery disease are likely parallel expressions of shared systemic processes, including atherosclerosis, aging, and cardiometabolic risk factors, rather than a direct cause–effect relationship [27,28].

The most prominent finding was the progressive and statistically significant increase in EFT with the angiographic severity of CAD. Mean EFT values increased stepwise from patients without significant CAD to those with one-vessel, two-vessel, and three-vessel disease, with strong statistical significance.

Our findings are consistent with previous echocardiographic and computed tomography studies demonstrating an association between epicardial fat thickness or volume and the presence and severity of coronary artery disease [28,29,30,31]. Importantly, the present study extends prior observations by demonstrating a relationship between epicardial fat thickness and functional ischemic burden assessed by dobutamine stress echocardiography. While prior studies have primarily focused on anatomical plaque burden, the present data support the relevance of epicardial fat thickness in relation to inducible myocardial ischemia.

When patients were stratified according to epicardial fat thickness (EFT > 5 mm vs. ≤5 mm), those with increased EFT were older, had higher body mass index, and were more frequently hypertensive or obese, all of which are well-established cardiovascular risk factors. More than two-thirds of patients with EFT > 5 mm exhibited significant CAD compared with approximately one-third of those with lower EFT values. This finding supports EFT as a simple, non-invasive imaging parameter associated with a higher likelihood of significant coronary stenosis. The observed threshold around 5–6 mm is consistent with prior echocardiographic studies that reported similar cut-off values (derived from receiver operating characteristic analyses) for the identification of angiographically significant CAD. Although the present study was not designed to define an optimal cut-off value, these findings reinforce the clinical relevance of this widely reported threshold [32,33,34,35].

Beyond anatomical disease, the present study demonstrates an association between EFT and functional parameters derived from dobutamine stress echocardiography. Patients with higher EFT exhibited significantly higher peak-stress wall WMSI, indicating a greater ischemic burden manifested as more extensive inducible left ventricular wall motion abnormalities. Although the overall prevalence of ischemia-positive stress echocardiography did not differ significantly between EFT groups, the higher WMSI observed in patients with increased EFT suggests that epicardial fat is related to the severity of ischemia rather than its mere presence.

While previous studies have primarily focused on the association between epicardial adipose tissue and angiographic measures of coronary plaque burden, relatively few have examined its relationship with functional ischemic assessment [36,37]. In this context, the present findings extend existing evidence by demonstrating an association between increased EFT and worse stress-induced left ventricular wall motion abnormalities. This observation is consistent with data from large clinical studies showing that the extent and severity of inducible ischemia, rather than anatomical stenosis alone, play a central role in guiding clinical decision-making [38,39,40]. Accordingly, functional markers such as peak-stress WMSI provide important diagnostic and prognostic information, supporting the potential value of combining EFT assessment with stress echocardiography.

From a clinical perspective, measurement of EFT during routine transthoracic echocardiography may provide incremental information for non-invasive risk stratification in patients with suspected coronary artery disease. Increased EFT may help identify patients who warrant closer functional evaluation, while lower values combined with negative stress testing may support conservative management strategies. The simplicity and reproducibility of EFT measurement make it an attractive adjunct to standard echocardiographic assessment.

Several limitations should be acknowledged. This was a single-center study with a moderate sample size, which may limit the generalizability of the findings. The study population was characterized by advanced age and a relatively high body mass index, both of which are strong and independent predictors of coronary artery disease. Consequently, the observed association between epicardial fat thickness (EFT) and coronary artery disease may, at least in part, reflect shared cardiovascular risk factors rather than an independent effect of epicardial adiposity.

Other anatomical and structural factors may also influence coronary artery disease risk. Previous studies have suggested that thoracic geometry, including a reduced antero–posterior thoracic diameter, may be associated with a lower prevalence of obstructive coronary artery disease and may influence epicardial fat distribution [41,42]. However, assessment of thoracic geometry was beyond the scope of the present study and warrants dedicated investigation in future research.

Furthermore, inflammatory and metabolic biomarkers related to epicardial adipose tissue activity, such as cytokines and adipokines, were not assessed, limiting mechanistic interpretation of the observed associations. The influence of epicardial adiposity in younger individuals or in patients with normal body mass index also remains uncertain. Future multicenter studies with larger populations, standardized EFT measurement protocols, and longitudinal follow-up with hard cardiovascular outcomes are required to further clarify the clinical and prognostic significance of epicardial fat thickness.

## 5. Conclusions

This study shows that EFT, measured non-invasively by transthoracic echocardiography, is significantly associated with both the severity of CAD and stress-induced myocardial dysfunction during DSE. EFT increased progressively with severity of CAD and correlated with the extent of ischemic burden at peak stress, manifested as wall motion disturbances. These findings suggest that EFT represents a reliable anatomical marker with direct functional relevance. These findings reflect an association between epicardial fat thickness and coronary artery disease and should be interpreted in the context of established cardiovascular risk factors, including age and body mass index. Thus, incorporating EFT into standard echocardiographic protocols may improve early identification of symptomatic patients who are likely to have significant CAD, guide decision making for further testing or revascularization, and refine non-invasive risk stratification. Future multicenter studies with larger cohorts, longitudinal follow-up, and standardized EFT measurement protocols are warranted to confirm the prognostic value and clinical utility of EFT in guiding therapeutic decisions.

## Figures and Tables

**Table 1:** Risk factors comparison of the EFT Groups. EFT: Epicardial Fat Thickness; BMI: Body Mass Index; PAD: Peripheral Artery Disease.

Variable	EFT > 5 mm (n = 80)	EFT ≤ 5 mm (n = 45)	*p*-Value
Age (years)	68.8 ± 8.9	62.0 ± 10.1	0.001
BMI	30.5 ± 4.3	27.8 ± 3.8	0.002
Hypertension	58 (72.5%)	20 (44.4%)	0.002
Dyslipidemia	55 (68.8%)	25 (55.6%)	0.133
Diabetes	24 (30.0%)	10 (22.2%)	0.336
Smoking	38 (47.5%)	22 (48.9%)	0.876
Obesity (BMI > 30)	33 (86.8%)	12 (48.0%)	0.001
PAD	10 (12.5%	5 (11.1%	0.815

**Table 2:** Comparison of significant CAD vs. no significant CAD. CAD: Coronary Artery Disease; EFT: Epicardial Fat Thickness; BMI: Body Mass Index; EF: Ejection Fraction; PAD: Peripheral Artery Disease.

Variable	Significant CAD n = 70	Non-Significant CAD n = 55	*p*-Value
Age (years, mean ± SD)	67.8 ± 9.1	64.0 ± 10.2	0.024
EFT (mm, mean ± SD)	7.8 ± 2.0	5.5 ± 1.5	<0.001
BMI (mean ± SD)	30.0 ± 4.3	28.8 ± 4.0	0.119
EF (%, mean ± SD)	52.8 ± 7.1	55.5 ± 6.2	0.022
Hypertension (n, %)	50 (71.4%)	28 (50.9%)	0.017
Dyslipidemia (n, %)	50 (71.4%)	30 (54.5%)	0.052
Diabetes (n, %)	22 (31.4%)	12 (21.8%)	0.234
Smoking (n, %)	35 (50.0%)	25 (45.5%)	0.612
PAD (n, %)	10 (14.3%)	5 (9.1%)	0.410

**Table 3:** The mean EFT values according to number of diseased CAs.

CAD Severity	Number of Patients	Mean EFT (mm) ± SD
Non-significant CAD	55	5.5 ± 1.5
1-vessel disease	30	6.5 ± 1.8
2-vessel disease	29	7.5 ± 2.0
3-vessel disease	11	8.5 ± 1.9

## Data Availability

The data presented in this study are not publicly available due to privacy and ethical restrictions but may be available from the corresponding author upon reasonable request and with appropriate approvals.

## Abbreviations

The following abbreviations are used in this manuscript:

EFT	Epicardial fat thickness
CAD	Coronary artery disease
WMSI	Wall motion score index
ACS	Acute coronary syndrome
PCI	Percutaneous coronary intervention
CABG	Coronary artery bypass graft
BMI	Body mass index
DSE	Dobutamine Stress Echocardiography
LAD	Left anterior descending artery
LCX	Left circumflex artery
RCA	Right coronary artery
LVEF	Left ventricular ejection fraction
TTE	Transthoracic echocardiograms

## References

[1] Villasante Fricke A.C., Iacobellis G. (2019). Epicardial Adipose Tissue: Clinical Biomarker of Cardio-Metabolic Risk. Int. J. Mol. Sci..

[2] Nagy E., Jermendy A.L., Merkely B., Maurovich-Horvat P. (2017). Clinical importance of epicardial adipose tissue. Arch. Med. Sci..

[3] Wu Y., Zhang A., Hamilton D.J., Deng T. (2017). Epicardial Fat in the Maintenance of Cardiovascular Health. Methodist Debakey Cardiovasc. J..

[4] Li C., Liu X., Adhikari B.K., Chen L., Liu W., Wang Y., Zhang H. (2023). The role of epicardial adipose tissue dysfunction in cardiovascular diseases: An overview of pathophysiology, evaluation, and management. Front. Endocrinol..

[5] Iacobellis G. (2021). Aging Effects on Epicardial Adipose Tissue. Front. Aging.

[6] Talman A.H., Psaltis P.J., Cameron J.D., Meredith I.T., Seneviratne S.K., Wong D.T. (2014). Epicardial adipose tissue: Far more than a fat depot. Cardiovasc. Diagn. Ther..

[7] Chaowalit N., Somers V.K., Pellikka P.A., Rihal C.S., Lopez-Jimenez F. (2006). Subepicardial adipose tissue and the presence and severity of coronary artery disease. Atherosclerosis.

[8] Milanese G., Silva M., Ledda R.E., Goldoni M., Nayak S., Bruno L., Rossi E., Maffei E., Cademartiri F., Sverzellati N. (2020). Validity of epicardial fat volume as biomarker of coronary artery disease in symptomatic individuals: Results from the ALTER-BIO registry. Int. J. Cardiol..

[9] Tanami Y., Jinzaki M., Kishi S., Matheson M., Vavere A.L., Rochitte C.E., Dewey M., Chen M.Y., Clouse M.E., Cox C. (2015). Lack of association between epicardial fat volume and extent of coronary artery calcification, severity of coronary artery disease, or presence of myocardial perfusion abnormalities in a diverse, symptomatic patient population: Results from the CORE320 multicenter study. Circ. Cardiovasc. Imaging.

[10] Yin R., Tang X., Wang T., Shi H., Wang X., Wang X., Pan C. (2020). Cardiac CT scanning in coronary artery disease: Epicardial fat volume and its correlation with coronary artery lesions and left ventricular function. Exp. Ther. Med..

[11] Molnar D., Björnson E., Hjelmgren O., Adiels M., Bäckhed F., Bergström G. (2025). Coronary artery calcifications are not associated with epicardial adipose tissue volume and attenuation on computed tomography in 1,945 individuals with various degrees of glucose disorders. Int. J. Cardiol. Heart Vasc..

[12] Chen Y.C., Lee W.H., Lee M.K., Hsu P.C., Tsai W.C., Chu C.Y., Lee C.S., Yen H.W., Lin T.H., Voon W.C. (2020). Epicardial adipose tissue thickness is not associated with adverse cardiovascular events in patients undergoing haemodialysis. Sci. Rep..

[13] Mahabadi A.A., Lehmann N., Kälsch H., Robens T., Bauer M., Dykun I., Budde T., Moebus S., Jöckel K.H., Erbel R. (2014). Association of epicardial adipose tissue with progression of coronary artery calcification is more pronounced in the early phase of atherosclerosis: Results from the Heinz Nixdorf recall study. JACC Cardiovasc. Imaging.

[14] Picard F.A., Gueret P., Laissy J.P., Champagne S., Leclercq F., Carrié D., Juliard J.M., Henry P., Niarra R., Chatellier G. (2014). Epicardial adipose tissue thickness correlates with the presence and severity of angiographic coronary artery disease in stable patients with chest pain. PLoS ONE.

[15] Filtz A., Lorenzatti D., Scotti A., Piña P., Fernandez-Hazim C., Huang D., Ippolito P., Skendelas J.P., Kuno T., Rodriguez C.J. (2024). Relationship between epicardial adipose tissue and coronary atherosclerosis by CCTA in young adults (18–45). Am. J. Prev. Cardiol..

[16] Bachar G.N., Dicker D., Kornowski R., Atar E. (2012). Epicardial adipose tissue as a predictor of coronary artery disease in asymptomatic subjects. Am. J. Cardiol..

[17] Eroglu S., Sade L.E., Yildirir A., Bal U., Ozbicer S., Ozgul A.S., Bozbas H., Aydinalp A., Muderrisoglu H. (2009). Epicardial adipose tissue thickness by echocardiography is a marker for the presence and severity of coronary artery disease. Nutr. Metab. Cardiovasc. Dis..

[18] Lang R.M., Badano L.P., Mor-Avi V., Afilalo J., Armstrong A., Ernande L., Flachskampf F.A., Foster E., Goldstein S.A., Kuznetsova T. (2015). Recommendations for cardiac chamber quantification by echocardiography in adults: An update from the American Society of Echocardiography and the European Association of Cardiovascular Imaging. J. Am. Soc. Echocardiogr..

[19] Galderisi M., Cosyns B., Edvardsen T., Cardim N., Delgado V., Di Salvo G., Donal E., Sade L.E., Ernande L., Garbi M. (2017). Standardization of adult transthoracic echocardiography reporting in agreement with recent chamber quantification, diastolic function, and heart valve disease recommendations: An expert consensus document of the European Association of Cardiovascular Imaging. Eur. Heart J. Cardiovasc. Imaging.

[20] Picano E., Pierard L., Peteiro J., Djordjevic-Dikic A., Sade L.E., Cortigiani L., Van De Heyning C.M., Celutkiene J., Gaibazzi N., Ciampi Q. (2024). The clinical use of stress echocardiography in chronic coronary syndromes and beyond coronary artery disease: A clinical consensus statement from the European Association of Cardiovascular Imaging of the ESC. Eur. Heart J. Cardiovasc. Imaging.

[21] Pellikka P.A., Arruda-Olson A., Chaudhry F.A., Chen M.H., Marshall J.E., Porter T.R., Sawada S.G. (2020). Guidelines for Performance, Interpretation, and Application of Stress Echocardiography in Ischemic Heart Disease: From the American Society of Echocardiography. J. Am. Soc. Echocardiogr..

[22] Zhang P.F., Tian J.W., Zhu T.G., Wu J.F., Leng X.P., Wang Y., Li M.M., Li X.H., Wang Q.Q., Feng X.P. (2024). Stress Echocardiography for Chronic Coronary Syndrome: Clinical Practice Guidelines (2023). J. Geriatr. Cardiol..

[23] Iacobellis G., Bianco A.C. (2011). Epicardial adipose tissue: Emerging physiological, pathophysiological and clinical features. Trends Endocrinol. Metab..

[24] Salazar J., Luzardo E., Mejías J.C., Rojas J., Ferreira A., Rivas-Ríos J.R., Bermúdez V. (2016). Epicardial Fat: Physiological, Pathological, and Therapeutic Implications. Cardiol. Res. Pract..

[25] Iacobellis G. (2014). Epicardial adipose tissue in endocrine and metabolic diseases. Endocrine.

[26] Shimabukuro M., Hirata Y., Tabata M., Dagvasumberel M., Sato H., Kurobe H., Fukuda D., Soeki T., Kitagawa T., Takanashi S. (2013). Epicardial adipose tissue volume and adipocytokine imbalance are strongly linked to human coronary atherosclerosis. Arterioscler. Thromb. Vasc. Biol..

[27] La V., Nair V., Sunny S., Benharash P., Thankam F.G. (2025). Epicardial Adipocytes in Cardiac Pathology and Healing. Cardiovasc. Drugs Ther..

[28] Sahasrabuddhe A.V., Pitale S.U., Sivanesan S.D., Deshpande P.K., Deshpande S.P., Daiwile A. (2020). Pathogenic gene expression of epicardial adipose tissue in patients with coronary artery disease. Indian J. Med. Res..

[29] Yu W., Chen Y., Zhang F., Liu B., Wang J., Shao X., Yang X., Shi Y., Wang Y. (2023). Association of epicardial adipose tissue volume with increased risk of hemodynamically significant coronary artery disease. Quant. Imaging Med. Surg..

[30] Zhou H., Pan Y., Du J., Liang F., Ma X., Lv D. (2025). Association of epicardial fat volume with the severity of coronary artery disease: A preliminary study on risk prediction of obstructive coronary heart disease. BMC Cardiovasc. Disord..

[31] Mohar D.S., Salcedo J., Hoang K.C., Kumar S., Saremi F., Erande A.S., Naderi N., Nadeswaran P., Le C., Malik S. (2014). Epicardial adipose tissue volume as a marker of coronary artery disease severity in patients with diabetes independent of coronary artery calcium: Findings from the CTRAD study. Diabetes Res. Clin. Pract..

[32] Braescu L., Sturza A., Sosdean R., Aburel O.M., Lazar M.A., Muntean D., Luca C.T., Brie D.M., Feier H., Crisan S. (2024). Echocardiographic assessment of epicardial adipose tissue thickness as independent predictor in coronary artery disease. Can. J. Physiol. Pharmacol..

[33] Akhter A.N., Aisha F., Moazzam A.B., Khan S.H.B., Malik J., Parveen A. (2025). Epicardial Fat Thickness as a Marker of Coronary Artery Disease in Diabetics: A Single Center Study. Clin. Cardiol..

[34] Shambu S.K., Desai N., Sundaresh N., Babu M.S., Madhu B., Gona O.J. (2020). Study of correlation between epicardial fat thickness and severity of coronary artery disease. Indian Heart J..

[35] Achim A., Szűcsborus T., Sasi V., Nagy F., Jambrik Z., Nemes A., Varga A., Homorodean C., Bertrand O.F., Ruzsa Z. (2022). Safety and Feasibility of Distal Radial Balloon Aortic Valvuloplasty: The DR-BAV Study. JACC Cardiovasc. Interv..

[36] Tamarappoo B., Dey D., Shmilovich H., Nakazato R., Gransar H., Cheng V.Y., Friedman J.D., Hayes S.W., Thomson L.E., Slomka P.J. (2010). Increased pericardial fat volume measured from noncontrast CT predicts myocardial ischemia by SPECT. JACC Cardiovasc. Imaging.

[37] Muthalaly R.G., Nerlekar N., Wong D.T., Cameron J.D., Seneviratne S.K., Ko B.S. (2017). Epicardial adipose tissue and myocardial ischemia assessed by computed tomography perfusion imaging and invasive fractional flow reserve. J. Cardiovasc. Comput. Tomogr..

[38] Boden W.E., O’Rourke R.A., Teo K.K., Hartigan P.M., Maron D.J., Kostuk W.J., Knudtson M., Dada M., Casperson P., Harris C.L. (2007). Optimal medical therapy with or without PCI for stable coronary disease. N. Engl. J. Med..

[39] Maron D.J., Hochman J.S., Reynolds H.R., Bangalore S., O’Brien S.M., Boden W.E., Chaitman B.R., Senior R., López-Sendón J., Alexander K.P. (2020). Initial Invasive or Conservative Strategy for Stable Coronary Disease. N. Engl. J. Med..

[40] Kanoun Schnur S.S., Achim A., Toth G.G. (2023). Clinical application of results of the ISCHEMIA trial. Trends Cardiovasc. Med..

[41] Sonaglioni A., Nicolosi G.L., Bruno A., Lombardo M., Muti P. (2024). Echocardiographic Assessment of Mitral Valve Prolapse Prevalence before and after the Year 1999: A Systematic Review. J. Clin. Med..

[42] Freed L.A., Levy D., Levine R.A., Larson M.G., Evans J.C., Fuller D.L., Lehman B., Benjamin E.J. (1999). Prevalence and clinical outcome of mitral-valve prolapse. N. Engl. J. Med..

